# Public Meeting of the Dental Profession

**Published:** 1856-12

**Authors:** 


*Public Meeting of the Dental Profession.*—On Monday evening last, a numerous and influential meeting of members of the dental profession and others interested in the advancement of dental science, was held at the London Tavern, Bishopsgate street, when resolutions in favor of establishing a Society of Dentists, similar in many respects to the existing medical societies, and of establishing a College of Dentistry, were unanimously carried. The meeting was very ably presided over by Mr. Alfred Carpenter, M. B., who, as a medical practitioner, could speak to the importance of the movement in an impartial manner. The resolutions were proposed and spoken to by Mr. S. L. Rymer, Mr. Thompson, Mr. Mackenzie, Mr. Peter Mathews, Mr. W. Perkins, &c. Remarkable unanimity characterized the whole proceedings, which were brought to a close (after the appointment of a committee of twenty-five to carry the resolutions into effect) by a cordial vote of thanks to the chairman.—*London Lancet*, Sept. 27th.

*Phosphate of Lime in the Treatment of Fractures.*—We notice in a late number of the *Gazette des Hopitaux*, some cases of fracture, in which the union of the bones appeared to be promoted by the administration of the phosphate of lime. In one of these cases, of fracture of the humerus, there was union in forty-five days, *without* the phosphate. The patient, a fortnight afterwards, fractured the arm in the same place, by a fall from a horse. The phosphate of lime was then prescribed, and the arm was placed in splints, as before; the bones united in *thirty-five* days. The man had the ill luck to break the callus a third time, and, under the use of the lime, the fracture was consolidated in *twenty-five* days. The remedy in question has long been employed by M. Piorry in the treatment of rickets, mollities ossium and Pott's disease, but it appears to have been only recently suggested by M. A. Milne Edwards as a useful remedy in cases of fracture. We are surprised that no allusion is made to its employment in ununited fracture; whether it has been tried in these cases, which are often so difficult to cure, we do not know; it would seem that it could hardly fail to be of service.

